# Characterization of Influenza A Virus Infection in Mouse Pulmonary Stem/Progenitor Cells

**DOI:** 10.3389/fmicb.2019.02942

**Published:** 2020-01-21

**Authors:** Tai-Ling Chao, Sing-Yi Gu, Pi-Han Lin, Yu-Tien Chou, Thai-Yen Ling, Sui-Yuan Chang

**Affiliations:** ^1^Department of Clinical Laboratory Sciences and Medical Biotechnology, National Taiwan University College of Medicine, Taipei, Taiwan; ^2^Department of Pharmacology, National Taiwan University College of Medicine, Taipei, Taiwan; ^3^Department of Laboratory Medicine, National Taiwan University Hospital, Taipei, Taiwan

**Keywords:** influenza, influenza A virus, mouse pulmonary stem cells, mouse pulmonary progenitor cells, pro-inflammatory responses

## Abstract

The pulmonary stem/progenitor cells, which could be differentiated into downstream cells to repair tissue damage caused by influenza A virus, have also been shown to be the target cells of influenza virus infection. In this study, mouse pulmonary stem/progenitor cells (mPSCs) with capability to differentiate into type I or type II alveolar cells were used as an *in vitro* cell model to characterize replication and pathogenic effects of influenza viruses in PSCs. First, mPSCs and its immortalized cell line mPSCs^Oct4+^ were shown to be susceptible to PR8, seasonal H1N1, 2009 pandemic H1N1, and H7N9 influenza viruses and can generate infectious virus particles, although with a lower virus titer, which could be attributed by the reduced vRNA replication and nucleoprotein (NP) aggregation in the cytoplasm. Nevertheless, a significant increase of interleukin (IL)-6 and interferon (IFN)-γ at 12 h and IFN-β at 24 h post infection in mPSCs implicates that mPSCs might function as a sensor to modulate immune responses to influenza virus infection. In summary, our results demonstrated mPSCs, as one of the target cells for influenza A viruses, could modulate early proinflammatory responses to influenza virus infection.

## Introduction

Influenza virus can cause acute and severe respiratory diseases. According to the WHO, about 3–5 million people are severely infected with influenza virus, and among those, 290,000–650,000 people die annually (World Health Organization [WHO], 2017). Compared to most seasonal influenza viruses, pandemic influenza viruses prefer to infect lower respiratory airways and often cause severe lung disease, such as pneumonia and acute respiratory distress syndrome (ARDS) (Kim et al., 2002). The lower respiratory tract consists of the trachea, bronchi, bronchioles, and alveoli. Alveoli are the ends of the respiratory tree and act as the basic units of ventilation of the lung. The alveoli consist of an epithelial layer for gas exchange, in which two types of cells, alveolar type-I pneumocytes (AT-I) and type-II pneumocytes (AT-II), are there. AT-I is responsible for gas exchange, whereas AT-II maintains surface tension of alveolus through secretion of surfactant proteins, such as SP-A, SP-B, SP-C, and SP-D (Whitsett and Alenghat, 2015). Previous studies have shown that human AT-I (Chan et al., 2009; Yu et al., 2011), human AT-II (Chan et al., 2005), murine AT-I (Kebaabetswe et al., 2013; Rosenberger et al., 2014), and murine AT-II (Kebaabetswe et al., 2013) can be infected by influenza A viruses. High-level expression of cytokines, such as interferon (IFN)-β, interleukin (IL)-6, or IL-1β, and chemokines, like RANTES, IFN-γ-induced protein 10 (IP-10), and macrophage inflammatory protein 1β (MIP-1β), was reported in infected AT-I and AT-II cells. However, little information for the outcomes of lung stem/progenitor cells by the infection of influenza virus A has been reported.

Upon influenza virus infection, the adult stem/precursor cells can proliferate and differentiate into various downstream cells to repair the damage in lung and even function as immune regulators (Park et al., 2010; Maul et al., 2011; Volckaert and De Langhe, 2014; Branchfield et al., 2016). Among the many different pulmonary stem/progenitor cells (PSCs) in the respiratory system, AT-II (Barkauskas et al., 2013) and bronchioalveolar stem cells (BASC) (Kim et al., 2005) have been reported as potential adult lung stem/progenitor cells. Recent studies in the mouse model demonstrated that influenza virus infection could induce the migration and differentiation of the upper respiratory tract’s stem/progenitor epithelial cells to compensate for damage in the lower respiratory tract (Kumar et al., 2011; Zuo et al., 2015). Another study also found that club cells could differentiate into AT-I and AT-II to repair impaired alveolar regions after virus infection (Zheng et al., 2012). To investigate the importance of pulmonary stem/precursor cells in influenza virus infection, adult PSCs have been isolated from different animal models. Chicken lung mesenchymal stromal cells have been shown to be infected by avian H9N5 and H1N1 influenza strain (Khatri et al., 2010). Moreover, the Oct4^+^ swine stem/progenitor lung epithelial cells could be infected by human, swine, and avian influenza viruses (Khatri et al., 2012). In C57BL/6 mice, influenza virus was identified to block the self-renewal of pulmonary epithelial stem/progenitor cells through blockage of fibroblast growth factor receptor 2b (Fgfr2b) signaling pathway (Quantius et al., 2016). Therefore, *via* these findings, we hypothesized that pulmonary stem/progenitor epithelial cells may not only be responsible for repair of lung tissues but also be one of the important target cells for influenza virus infection.

In this study, we aimed to demonstrate the infection of influenza virus to PSCs and try to establish the mouse pulmonary stem/progenitor epithelial cell lines to characterize the influences of influenza virus infection in pulmonary stem/progenitor epithelial cells. Previously, we have identified one rare population of mouse pulmonary stem/progenitor cells (named mPSCs) (Ling et al., 2006). The mPSCs could proliferate to form epithelial cell colonies accompanied by stroma cells cocultured in a serum-free culture condition and expressed in low level of pluripotent transcriptional factors (e.g., Oct4, Sox-2, and Nanog) and could differentiate into type-I pneumocytes *in vitro* (Ling et al., 2006, 2014). Our studies also demonstrated that mPSCs expressed a specific cellular surface marker, coxsackievirus/adenovirus receptor (CAR), which could be applied as a selective marker to isolate mPSCs (CAR^+^/mPSCs) from the culture for pure population by fluorescence-activated cell sorting (FACS) technique (Ling et al., 2014). In this study, we first demonstrated that mPSCs were also susceptible to influenza virus infection and then immortalized CAR^+^/mPSCs (mPSCs^Oct4+^ cell lines: G2L, E3L, and G4L clones) through overexpression of the pluripotent transcription factor, Oct-4, by retroviruses. The mPSCs^Oct4+^ cell lines were also susceptible to infections of the mouse-adapted influenza virus PR8 strain, seasonal H1N1, pandemic H1N1, and H7N9 influenza A viruses, with a lower efficiency of virus replication, which could be possibly due to impaired vRNA replication in mPSCs^Oct4+^ and irregular distribution of nucleoprotein (NP) proteins during viral replication. Finally, elevated expression of pro-inflammatory cytokines including IFN-β, IFN-γ, IL-6, IL-12p40p70, monocyte chemotactic protein 5 (MCP5), stem cell factor (SCF), soluble tumor necrosis factor receptor I (sTNFRI), and vascular endothelial growth factor (VEGF) was detected in PR8-infected cells at 12 h post infection (hpi) compared to mock cells. Our results implicated that mPSCs were susceptible to influenza virus infection and could trigger host defense through pro-inflammatory cytokines releasing. The immortalized mPSCs have the potential to serve as an *in vitro* model to examine the pathogenesis of influenza virus infection in mouse lungs.

## Materials and Methods

### Cells

Madin-Darby canine kidney (MDCK) cells (ATCC^®^ CCL-34^TM^) were grown in minimal essential medium (MEM; 61100-61, Gibco, United States) supplemented with 10% fetal bovine serum (FBS; 10082-147, Gibco, United States), penicillin G sodium 100 units/ml, streptomycin sulfate 100 μg/ml, and amphotericin B 250 ng/ml (antibiotic-antimycotic; 15240-062, Gibco, United States). Human renal cell line HEK293T cells (ATCC^®^ CRL-3216^TM^) were grown in Dulbecco’s modified Eagle medium (DMEM; 12100-046, Gibco, United States) supplemented with 10% FBS and antibiotics as described above.

### Virus Infection

The viruses used in this study included laboratory strain PR8 virus [A/Puerto Rico/8/34 (H1N1)], 2009 pandemic H1N1 strain [A/California/07/2009 (H1N1)], 2009 seasonal H1N1 clinical strains, 2016 pandemic H1N1 clinical strains, and H7N9 clinical strains [A/Taiwan/S02076/2013 (H7N9)]. Laboratory strain PR8 virus was generated by the reverse genetic system from Dr. R. G. Webster (Hoffmann et al., 2000). The virus titers were determined by plaque assay to calculate the multiplicity of infection (MOI) used for infection. For virus infection, the supernatants of cell culture were first removed, and the cells were incubated with virus input for 1 h. After that, the cells were washed with phosphate-buffered saline (PBS) before fresh medium was added into cell cultures. Culture supernatant was harvested at indicated times.

### Isolation of Mouse Primary Stem Cells

Newborn ICR mice were obtained from National Laboratory Animal Center and National Applied Research Laboratories (Taipei, Taiwan). Primary pulmonary cells were isolated as described previously (Ling et al., 2006). Briefly, lung tissue was collected from 0 to 2 days postpartum newborn ICR mice. After washing with Hank’s buffer, tissue was cut into 5-mm pieces and digested with 10 mg/mL protease in Joklik’s MEM medium (M8028, Sigma-Aldrich, United States) at 4°C for 16 h. Tissues were transferred into 10% FBS Joklik’s MEM medium and filtered through a 100-μm nylon cell strainer. These cells were washed and resuspended in MCDB-201 medium (M6770, Sigma-Aldrich, United States). The cells were plated in collagen I (10 μg/cm^2^; 354236, BD Bioscience, United States) coated plates in MCDB-201 medium containing 1% Insulin-Transferrin-Selenium-A supplement (ITS-A, 51300-044, Gibco, United States), 1 ng/ml human EGF (PHG0311L, Gibco, United States), and 1% antibiotic solution (15240-062, Gibco, United States). After removal of the non-adherent cells after 48 h, cultures were maintained before further experiments. The detailed protocol had been described in previous studies (Ling et al., 2006, 2014).

### Immortalization of mPSCs by Oct4 Overexpression Using Retrovirus Transduction

Mouse PSCs were transduced with retroviral vectors encoding Oct4. Briefly, pMX-mOct4 retroviral vector and VSV-G were transfected into HEK293T cells using TurboFect transfection reagent (R0532, Thermo Scientific, United States). After 48 h, virus supernatants were collected and filtrated through a 0.45-μm filter. A total of 5 × 10^4^ mPSCs were seeded per well in the six-well plate and then incubated with harvested retroviruses and 8 μg/ml polybrene for 24 h. The transduced mPSCs were then cocultured with inactivated MEFs with fresh murine embryonic stem (mES)/MCDB201 (1:1) medium added every day. Colony formation of transduced mPSCs was observed after 28 days of induction (Gu et al., 2016). Different to the previous study (Gu et al., 2016), the colonies were picked up and expanded on the collagen I coating plate. The cells of mPSCs^Oct4+^ G2L, E3L, and G4L clones were cultured in ES/MCDB201 medium and consecutively cultured for more than 20 passages.

### Immunofluorescence

The cells were fixed in 4% paraformaldehyde for 10 min, then penetrated with 0.1% triton in PBS. Non-specific binding was blocked using 3% bovine serum albumin in PBS for 1 h at room temperature. Cells were then incubated at 4°C overnight or at 37°C for 90 min with the primary antibodies. The primary antibodies used include anti-influenza M1 protein antibody (GTX40910, Gene Tex Incorporation, United States), anti-influenza NP protein antibody (GTX125989, Gene Tex Incorporation, United States), anti-influenza PB2 antibody (GTX125926, Gene Tex Incorporation, United States), anti-influenza PB1 antibody (GTX125923, Gene Tex Incorporation, United States), anti-influenza PA antibody (GTX118991, Gene Tex Incorporation, United States), anti-CAR antibody (AF2654, R&D Systems, United States), anti-CD54 antibody (AF796, R&D Systems, United States), anti-Oct4 antibody (sc-5279, Santa Cruz Biotechnology, United States), anti-Sox2 antibody (MAB2018, R&D Systems, United States), anti-*E*-cadherin antibody (610181, BD BioScience, United States), and anti-SPC antibody (AB3768, Millipore, United States). After incubation, cells were washed and incubated for 1 h at room temperature with the respective secondary antibodies, including cyanine 3 (Cy3)-labeled goat anti-mouse IgG (115-165-003, Jackson ImmunoResearch, United States, 1:500), Cy3-labeled rabbit anti-goat IgG (705-165-147, Jackson ImmunoResearch, United States, 1:500), Alex488-labeled rabbit anti-goat IgG, and fluorescein isothicyanate (FITC)-labeled goat anti-rabbit IgG (111-095-003, Jackson ImmunoResearch, United States, 1:500). The nuclei of cells were counterstained with DAPI (D9542, Sigma-Aldrich, United States) for another 20 min at room temperature. The expression of proteins was observed by fluorescence microscope (IX-71, Olympus).

### Flow Cytometry

The expression of sialic acids, CAR, and CD54 on mPSCs and mPSCs^Oct4+^ cells was detected by flow cytometry. The expressions of α2,6-linked sialic acid (α2,6 SA) and α2,3-linked sialic acid (α2,3 SA) were identified by fluorescein *Sambucus nigra* bark lectin (SNA, FL-1301, Vector Laboratories, United States) or biotinylated *Maackia amurensis* lectin II (MALII, B-1265, Vector Laboratories, United States) with Cy^TM^3 Streptavidin (016-160-084, Jackson ImmunoResearch, United States). CD54 and CAR expressions were detected by PE-conjugated anti-CD54 IgG (116107, Biolegend, United States) and Alex488-conjugated anti-CAR IgG (bs2839R, Bioss, United States). Data acquired from Accuri C6 flow cytometer (BD Accuri, United States) were analyzed using CFlow plus software (BD Accuri, United States).

### Quantification of Cellular mRNA and Viral vRNA, mRNA, and cRNA

The total RNA of mock or virus-infected MDCK and mPSCs^Oct4+^ was extracted with NucleoSpin^®^ RNA kit (740955, MACHEREY-NAGAL, Germany) according to the manufacturer’s instructions. Complementary DNA of cellular and three viral RNA were synthesized using SuperScript^TM^ III reverse transcriptase (18080-044, Invitrogen, United States). The real-time PCR reaction contains 5 μl of cDNA, 300 nM of each primer, and 10 μl Fast SYBR^®^ Green Master mix (4385612, Applied Biosystems, United States). The PCR program consists of incubation at 95°C for 20 s, followed by 40 cycles of 3 s at 95°C and 30 s at 60°C. The universal and real-time PCR primer sequences are listed in the Supplementary Table S1. Data analysis was performed using Applied Biosystems 7500 Real-Time PCR software (version 7500SDS v1.5.1). At least three independent experiments were performed for all data presented. The relative expression of RNA was normalized with its internal control *GAPDH*, and a fold change relative to the normalized value at 0 hpi was shown.

### Cytokines and IFN-β Expression Detection

A total of 8 × 10^5^ mPSCs^Oct4+^ cells were infected with 10 MOI of PR8, and at 12 hpi, cells and culture supernatants were collected for protein expression and cytokine detection, respectively. The cytokine profiles were determined by the mouse cytokine antibody arrays (ab133993, Abcam, United Kingdom) according to the manufacturer’s instructions. Briefly, the supplied membranes were blocked with provided blocking buffer for 45 min and then incubated at 4°C overnight with 1 ml of harvested supernatants. Membranes were washed with the provided buffers and incubated at 4°C overnight with the supplied biotin-conjugated anti-cytokines antibodies. The arrays were then washed, incubated with horseradish peroxidase-conjugated streptavidin at 4°C overnight. After a final wash, chemiluminescence reaction was detected using the supplied detection buffers. Semiquantification by relative densitometry was obtained using the Image Lab^TM^ vision 6.0 (Bio-Rad, United States) and normalized to the positive control signals in each membrane for comparison of multiple arrays. The cytokine expression in virus-infected mPSCs^Oct4+^ and mock-infected cells was normalized to their respective internal controls, then the fold change of cytokine expression in virus-infected mPSCs^Oct4+^ relative to mock-infected cells was calculated. For determination of IFN-β expression, the VeriKine-HS^TM^ mouse IFN beta serum ELISA Kit was used (42410, pbl Assay Science; United States).

### Statistical Analysis

All data are representative of at least three independent experiments. Data were expressed as mean ± SD. All statistical analyses were performed using SPSS (ver. 12.0, Chicago, IL, United States). A two-sample *t* test was used for the comparison of continuous variables, with correction of unequal variances when appropriate. A *p* < 0.05 was considered statistically significant.

## Supplementary Methods

### Transmission Electron Microscopy

The mPSCs, mPSCs^Oct4+^, and MDCK cells were infected with PR8 at the MOI of 10. At 12 hpi, the cells were trypsinized and resuspended in PBS. After centrifugation at 1,000 rpm for 5 min (Model 3300, Kubota, Japan), the cell pellets were collected and fixed with cold 10% glutaraldehyde for at least 1 h. Cell pellets were picked up and then dehydrated with graded ethanol solution (100983, Merck, Germany). Finally, cells were embedded in Spurr’s resin (EM0300, Sigma-Aldrich, United States), and the embedded cell blocks were cut into ultrathin sections. Twenty-five sections were collected per sample and put on copper grids. The cells were stained with 1% uranyl acetate (541093, Thomas Scientific, United States) and 1% lead citrate (C6522, Sigma-Aldrich, United States). The grids were then observed by the JEOL JEM-1400 electron microscope (JEOL, Japan). The experiments were facilitated by the electron microscopy service of the Joint Center for Instruments and Researches, College of Bio-resources and Agriculture, National Taiwan University, Taiwan.

### Sucrose Gradient Ultracentrifugation

MDCK and mPSCs^Oct4+^ cells were infected by PR8 at an MOI of 10. Cultured supernatant of virus-infected cells was harvested at 36 hpi and then clarified by centrifugation at 1,500 rpm for 5 min at room temperature. Viruses in clarified supernatant were concentrated by 20% sucrose ultracentrifugation at 20,000 rpm for 2 h at 4°C (SW28 rotor, Optima L100-K, Beckman Coulter, Inc., United States). Concentrated viruses were resuspended in 1 ml TNE buffer (50 mm Tris–HCl, pH 7.4, 100 mm NaCl, and 0.1 mm EDTA) and then were loaded onto 9-ml linear sucrose gradient (30–60% w/v). Ultracentrifugation was performed at 30,000 rpm for 16 h at 4°C (SW41 rotor, Optima L100-K, Beckman Coulter, Inc., United States). Ten or 20 fractions were taken from the gradients. Virus titers in each fraction were quantified by plaque assay.

### Western Blotting

Mouse PSCs and mPSCs^Oct4+^ were harvested and lysed with RIPA buffer. The cell lysates and viruses in fractions were subjected to 12% SDS-PAGE and then transferred to a PVDF membrane for Western blot analysis. Monoclonal rabbit anti-Oct4 antibody (sc-9081, Santa Cruz Biotechnology, United States 1:200), polyclonal anti-GAPDH antibody (GTX100118, Gene Tex International Corporation, United States, 1:1,000), polyclonal anti-NP antibody (GTX125989, Gene Tex International Corporation, United States, 1:10,000), and polyclonal anti-NS1 antibody (GTX125990, Gene Tex International Corporation, United States, 1:1,000) were used as the primary antibody, and HRP-conjugated anti-mouse IgG (626520, Invitrogen, United States, 1:5,000) and HRP-conjugated anti-rabbit IgG (G21234, Invitrogen, United States, 1:5,000) were used as the secondary antibody, respectively. The presence of proteins was detected by the Western Lightning^®^ Plus-ECL system (NEL105001EA, PerkinElmer, United States) and the ChemiDoc^TM^ XRS + system (Bio-Rad, United States). Data were analyzed by the Image Lab^TM^ software (vision 6.0, Bio-Rad, United States).

### Virus Binding, Penetration, and Entry Assay

MDCK and mPSCs^Oct4+^ cells were preincubated at 4°C for 30 min, and medium was replaced with serum-free DMEM before virus infection. The cells were infected by virus with an MOI of 10 at 4°C for 1 h. The supernatant containing unbound viruses was removed, and the cells were washed three times by PBS. The virus-bound cells were harvested for binding assay. For penetration assay, the cells were preincubated at 4°C for 30 min. After virus infection, the cells were incubated at 37°C for 1 h to allow virus penetration into the cell membrane. The cells were washed once by acidic PBS (pH 3.0) and twice by neutral PBS (pH 7.0) to remove virus, which did not penetrate into the cell membrane. For entry assay, the medium was replaced with serum-free DMEM, and the cells were infected by virus with an MOI of 10 at 37°C for 1 h. The supernatant containing unbound virus was removed, and the cells were washed once by acidic PBS (pH3.0) and twice by neutral PBS (pH 7.0) to remove viruses, which did not penetrate into the cell membrane. Viral RNA on/in the cells was extracted with NucleoSpin^®^ RNA kit (740955, MACHEREY-NAGAL, Germany) according to the manufacturer’s instructions. Viral RNA was determined by RT real-time PCR. The percentages of these assay were calculated as virus copies in/on the cells/virus input copies × 100%.

### Quantification of Virus Copies

Viral RNA was quantified by RT real-time PCR *via* iTaq^TM^ Universal Probes One-Step Kit (172-5140, Bio-Rad, United States). The real-time PCR reaction contains 5 μl viral RNA, 400 nM of each primer (INFA_1: 5′-GGA CTG CAG CGT AGA CGC TT-3′; INFA_2: 5′-CAT YCT GTT GTA TAT GAG GCC CAT-3′), 250 nM probe (INFA probe: 6FAM-CTC AGT TAT TCT GCT GGT GCA CTT GCC A-TAMRA), 10 μl iTaq universal probes reaction mix (2X), and 0.5 μl iScript reverse transcriptase. The PCR program consists of first incubation at 50°C for 10 min, then at 95°C for 1 min, followed by 40 cycles of 15 s at 95°C and 1 min at 60°C. Data analysis was performed using Applied Biosystems 7500 Real-Time PCR software (version 7500SDS v1.5.1). At least three independent experiments were performed for all data presented.

## Results

### Susceptibility of mPSCs to Influenza Virus Infection

**FIGURE 1:**
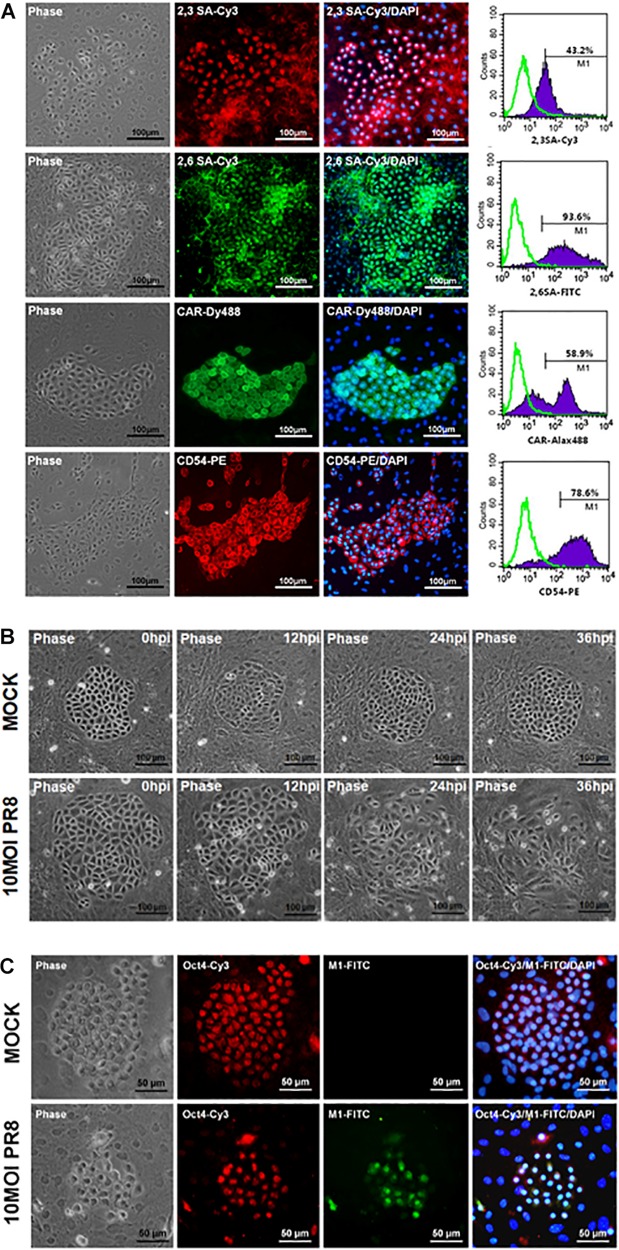
Infection of mouse pulmonary stem/progenitor cells (mPSCs) by influenza A virus. **(A)** The expression of α2,3-linked sialic acid (α2,3 SA) and α2,6-linked sialic acid (α2,6 SA) on mPSCs. The expression of α2,3 SA and α2,6 SA in mPSCs was determined by immunofluorescence assay (IFA) and fluorescence-activated cell sorting (FACS). The expression of CAR and CD54, which were previously demonstrated to be expressed by mPSCs, was also shown. The histograms of α2,3 SA, α2,6 SA, CAR, and CD54 expression of mPSCs were shown in purple, and the negative-staining cells were labeled as green lines. Scale bar of IFA image was 100 μm. **(B)** Development of cytopathic effects (CPEs) in mPSCs after influenza virus infection. mPSCs were infected with PR8 at a multiplicity of infection (MOI) of 10. The sequential changes in morphology of the same colony were recorded by microscope with a scale bar of 100 μm. **(C)** Colocalization of Oct4 and viral M1 proteins in influenza virus-infected mPSCs. The expression of Oct4 and viral M1 proteins in mPSCs at 24 h post infection (hpi) was determined by IFA. Scale bar was 50 μm.

Mouse PSCs were isolated from lung of neonatal ICR mice and further cultured for 7 days before virus infection. The primary mPSCs are surrounded by the mesenchymal stroma cells and have been shown to exhibit pulmonary stem/progenitor properties, including expression of progenitor markers and differentiation potential (Ling et al., 2006; Gu et al., 2016). High-level expression of influenza virus receptors, α2,3-linked sialic acid (α2,3 SA), and α2,6-linked sialic acid (α2,6 SA), was detected on mPSCs (Figure 1A). The expressions of CAR and CD54 on mPSCs were also confirmed by FACS (Figure 1A). The susceptibility of mPSCs to influenza virus infection was then examined. As shown in Figure 1B, the change of cell morphology and cytopathic effect (CPE) development were observed in the primary mPSCs upon influenza virus infection at an MOI of 10. Compared to the mock group, CPE was first observed at 12 hpi. The colony became less compact at 24 hpi and finally collapsed at 36 hpi. The infection was specific to mPSCs since the virus-infected cells were also costained with anti-M1 antibody and anti-Oct4 antibody (Figure 1C). Only mild cell morphology change at 24 hpi was observed when a lower virus input was used (Supplementary Figure S1). Infection of mPSCs by influenza virus was also confirmed using TEM (Supplementary Figure S2). Virus budding from influenza virus-infected mPSCs was observed, and the releasing virus particles had similar morphology to those released from MDCK cells. In addition, the susceptibility of mPSC-differentiated AT-I cells and AT-II cell line (MLE15) to influenza virus infection was also investigated. The CPE development and viral NP protein expression in AT-I cells and the AT-II cell line were observed at 24 hpi (Supplementary Figures S3A,B).

### Establishment and Characterization of Immortalized mPSCs Using Oct4 Overexpression

**FIGURE 2:**
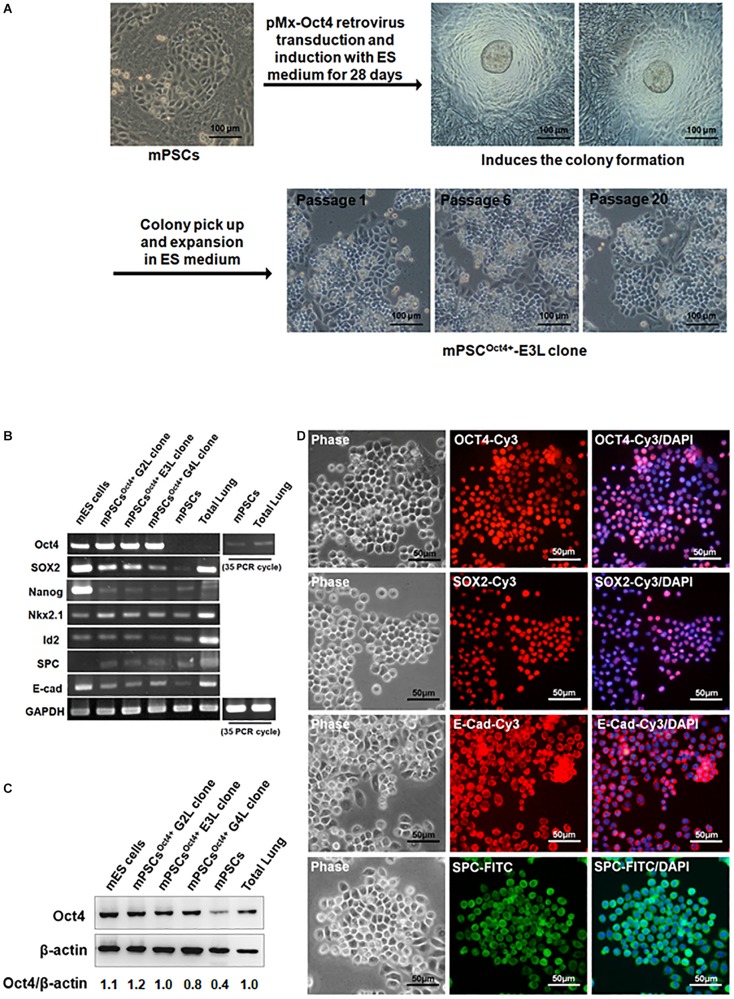
Generation and characterization of mouse pulmonary stem/progenitor cells (mPSCs)^Oct4+^, an Oct4-transduced immortalized mPSC cell line. **(A)** Experimental scheme for the establishment and selection of immortalized mPSCs. Scale bar was 100 μm. **(B)** Expression profiles of pulmonary progenitor/stem epithelial cell markers in mPSCs^Oct4+^ clones. Semiquantitative RT-PCR analysis was conducted to determine the expression of pulmonary progenitor/stem epithelial cell markers in mPSCs^Oct4+^ G2L, E3L, and G4L clones. The Oct4 expression in mPSCs and total lung was relatively lower and required up to 35 PCR cycles for visualized signals. Murine embryonic stem (mES) cells were used as a positive control for the stem cell markers, whereas extract from total lung was the positive control for pulmonary epithelial cell markers. **(C)** Expression of the OCT4 proteins in mPSCs^Oct4+^ G2L, E3L, and G4L clones. The expressions of OCT4 proteins in mPSCs^Oct4+^ G2L, E3L, and G4L clones were determined by Western blot. mES cells were used as a positive control. **(D)** Expression of OCT4, SOX2, E-Cad, and SPC in the mPSCs^Oct4+^ E3L clone by IFA. The expression of OCT4, SOX2, *E*-cadherin (*E*-cad), and Surfactant Protein C (SPC) in the mPSCs^Oct4+^ E3L clone was determined by IFA. Scale bar was 50 μm.

Due to the relatively low yield of isolating mPSCs from mouse lungs, an immortalized Oct4^+^ mPSCs cell line (mPSCs^Oct4+^) was established through transducing *Oct4* by retroviruses. The Oct4-transduced mPSCs gave rise to colonies after 28 days of incubation, and these colonies were picked up and expanded on collagen I-coated plates consecutively for more than 20 passages (Figure 2A). Among those, three colonies, designated mPSCs^Oct4+^ G2L, E3L, and G4L clones, were picked up and further analyzed. Increased Oct4 expression in mPSCs^Oct4+^ G2L, E3L, and G4L clones was observed by RT-PCR and Western blot, respectively (Figures 2B,C). In these cell lines, the expression level of the stem cell marker, Sox 2, was higher than that in mPSCs, yet the other stem cell marker, Nanog and pulmonary epithelial cell markers, Nkx2.1, Id2, SPC, and *E*-cadherin, had comparable expression levels to those in mPSCs (Figure 2B). The expression patterns of Oct4, Sox2, SPC, and E-cadherin in the mPSCs^Oct4+^ E3L clone were also demonstrated by the immunofluorescence analysis (Figure 2D). These results implicate that the mPSCs^Oct4+^ G2L, E3L, and G4L clones exhibited similar marker expression as those of mPSCs after immortalization by Oct4 overexpression.

### Susceptibility of mPSCs^Oct4+^ to Influenza Virus Infection

**FIGURE 3:**
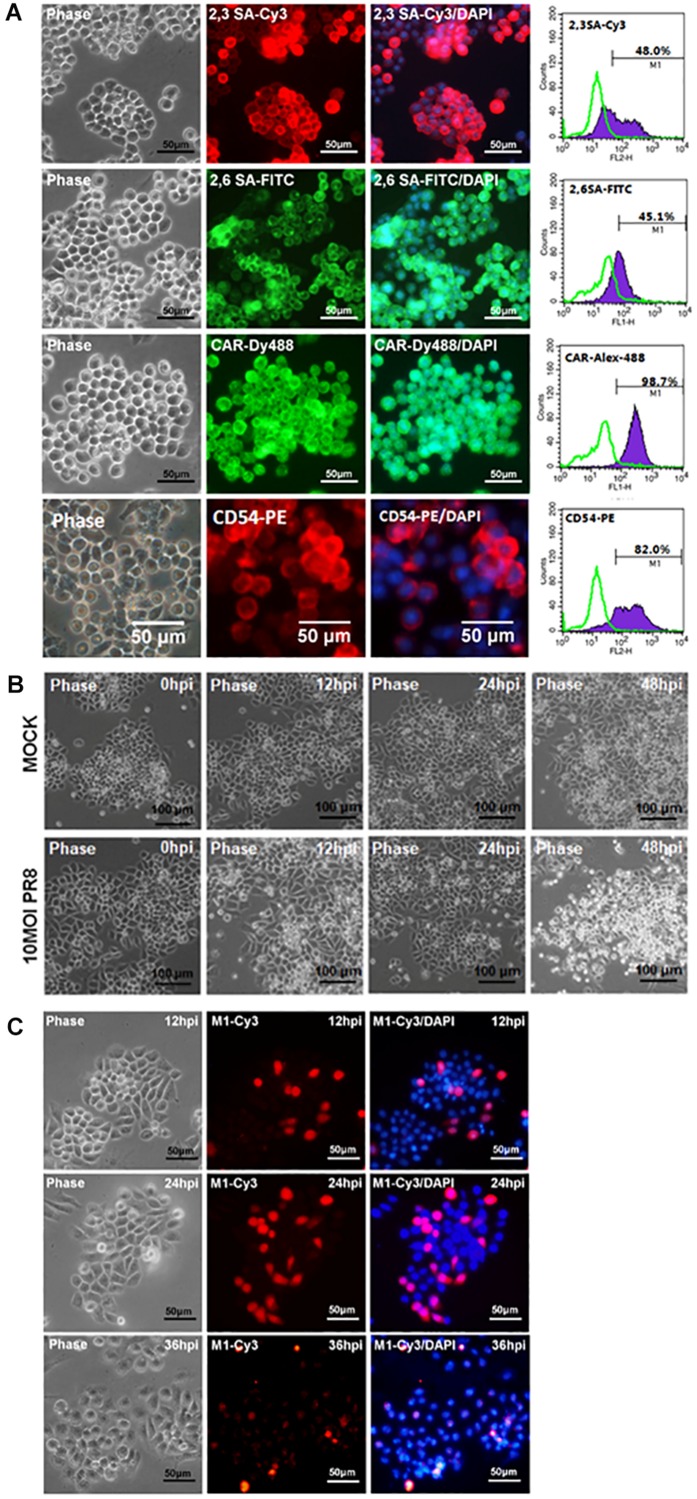
Infection of the mouse pulmonary stem/progenitor cells (mPSCs)^Oct4+^ E3L clone by influenza A virus. **(A)** The expression of α2,3-linked sialic acid (α2,3 SA) and α2,6-linked sialic acid (α2,6 SA) on the mPSCs^Oct4+^ E3L clone. The expression of α2,3 SA and α2,6 SA in mPSCs^Oct4+^ E3L clone was determined by immunofluorescence assay (IFA) and fluorescence-activated cell sorting (FACS). The expression of CAR and CD54, which was previously demonstrated to be expressed by mPSCs, was also shown. The histograms of α2,3 SA, α2,6 SA, CAR, and CD54 expression were shown in purple, and the negative-staining cells were labeled as green lines. Scale bar of IFA image was 50 μm. **(B)** Development of cytopathic effect (CPE) in the mPSCs^Oct4+^ E3L clone after influenza virus infection. The mPSCs^Oct4+^ E3L clone was infected with PR8 at a multiplicity of infection (MOI) of 10. The sequential changes in morphology were recorded by microscope with a scale bar of 100 μm. **(C)** Expression of viral M1 proteins in influenza virus-infected mPSCs^Oct4+^ E3L clone. The expression of viral M1 proteins in the mPSCs^Oct4+^ E3L clone at different time points after virus infection was determined by IFA. Scale bar was 50 μm.

The potential of Oct4-overexpressed immortalized mPSCs^Oct4+^ as an appropriate cell model for characterization of influenza virus infection of pulmonary epithelial cells was investigated. First, the mPSCs^Oct4+^ clones also expressed influenza virus receptors, α2,3 SA and α2,6 SA. The proportion of α2,3 SA- and α2,6 SA-positive cells were 48 and 45.1%, respectively, in the mPSCs^Oct4+^ E3L clone after 20 passages (Figure 3A). The expression of CAR and CD54 was also detected on the mPSCs^Oct4+^ E3L clone (Figure 3A). Sustained expression of sialic acid receptors on the mPSCs^Oct4+^ E3L clone after five to 20 passages was observed (Supplementary Figure S4). Next, the susceptibility of the mPSCs^Oct4+^ E3L clone to influenza virus infection was evaluated. Cells were infected by PR8 at an MOI of 10. A significant CPE induced by PR8 infection was observed at 48 hpi (Figure 3B). The expression of viral M1 proteins was detected in virus-infected cells at 12–48 hpi (Figure 3C). The release of virus particles from E3L clone was also confirmed using transmission electron microscope (TEM). The morphology of most virus particles was similar to those from MDCK and mPSCs cells (Supplementary Figure S2). The virus particles in the supernatant from virus-infected mPSCs^Oct4+^ E3L clone and MDCK cells were layered by linear sucrose gradient (30–60%) ultracentrifugation. To examine the distribution of virus in the gradients, viral infectivity and the amount of viral hemagglutinin (HA) and NP proteins were analyzed by plaque assay and Western blot, respectively, in all fractional samples. First, the density of viral particles from supernatants of MDCK and mPSCs^Oct4+^ E3L clone was estimated to be 1.20 and 1.18 g/ml, respectively (Supplementary Figure S5A). The distribution of HA and NP proteins was found to be consistent with that of virus titers across the gradient for mPSCs^Oct4+^ E3L clone but not for MDCK cells (Supplementary Figure S5B). More HA proteins were detected in fraction 3 instead of fraction 6 whose virus titer was highest.

### mPSCs^Oct4+^ E3L Clone Can Support Influenza Viruses’ Replication

**FIGURE 4:**
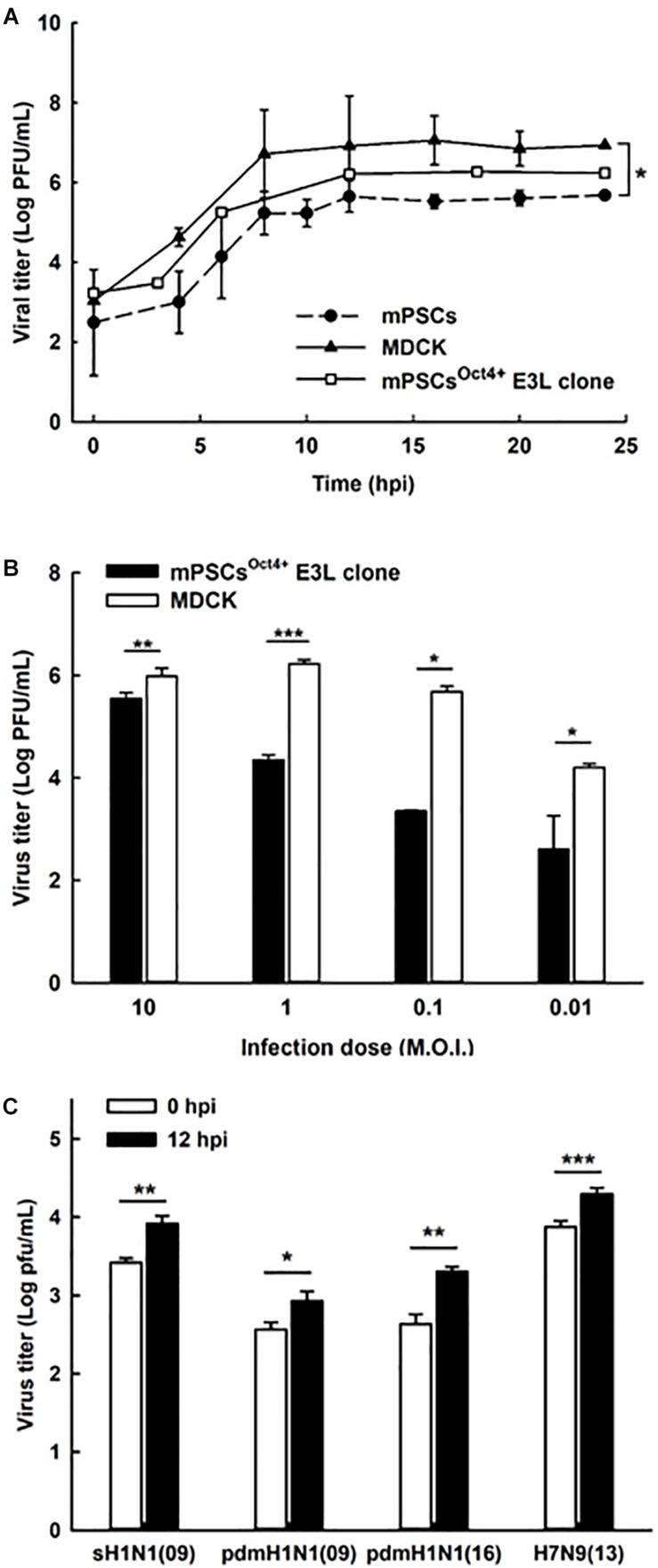
Replication of influenza viruses in mouse pulmonary stem/progenitor cells (mPSCs), mPSCs^Oct4+^ E3L clone, and Madin-Darby canine kidney (MDCK) cells. **(A)** Replication of influenza PR8 viruses in mPSCs, mPSCs^Oct4+^ E3L clone, and MDCK cells. Culture supernatants of mPSCs, mPSCs^Oct4+^ E3L clone, and MDCK cells were collected at indicated time points after virus infection, and the virus titers in the cultured supernatants were quantified by the plaque assay. **(B)** Comparison of influenza virus replication between mPSCs^Oct4+^ E3L clone and MDCK cells with different virus input. The mPSCs^Oct4+^ E3L clone and MDCK cells were infected with PR8 viruses at multiplicities of infection (MOIs) of 10, 1, 0.1, and 0.01. The virus titers at 12 hpi were determined by the plaque assay. **(C)** Replication of clinical influenza strains in mPSCs^Oct4+^ E3L clone. mPSCs^Oct4+^ E3L clone was infected with four human influenza virus strains, A/California/07/2009 (H1N1), A/Brisbane/59/2007 (H1N1)-like virus, A/Taipei/0056/2016(H1N1)-like virus, and A/Taiwan/S02076/2013 (H7N9) at an MOI of 10. The virus titers at 0 and 12 hpi were determined by the plaque assay. For the plaque assay, at least three independent experiments were performed, and the virus titers were presented as mean ± SD. ^∗^*p* < 0.05; ^∗∗^*p* < 0.01; ^∗∗∗^*p* < 0.001.

To characterize virus replication in mPSCs^Oct4+^ E3L clone, virus growth kinetics were determined. In the mPSCs^Oct4+^ E3L clone, virus growth was observed for 24 hpi, and it reached a plateau at 12 h (Figure 4A). Similar trend of virus replication was observed in mPSCs and MDCK when high virus input was used. Compared to MDCK cells, a reduced virus replication was observed when a lower virus input was used to infect the mPSCs^Oct4+^ E3L clone (Figure 4B). The virus growth kinetics in AT-I and MLE15 cells were also determined (Supplementary Figure S3C). Whereas viruses exhibited similar growth trend in AT-I cells as compared to the mPSCs and mPSCs^Oct4+^ E3L clone, a relatively lower virus growth was observed in MLE15 cells (Supplementary Figure S3C). The susceptibility of E3L clone to infection of other influenza subtypes was also determined. Among the four human influenza viruses, A/California/07/2009 (H1N1), A/Brisbane/59/2007 (H1N1)-like virus, A/Taipei/0056/2016(H1N1)-like virus, and A/Taiwan/S02076/2013 (H7N9), increased virus titers at 12 hpi were observed (Figure 4C). The specific expression of NP proteins in cells infected with these influenza viruses was also confirmed by immunofluorescence assay (IFA) (Supplementary Figure S6). These results implicate that the mPSCs^Oct4+^ E3L clone can support influenza virus replication.

### Life Cycle of Influenza Virus in E3L Clone

**FIGURE 5:**
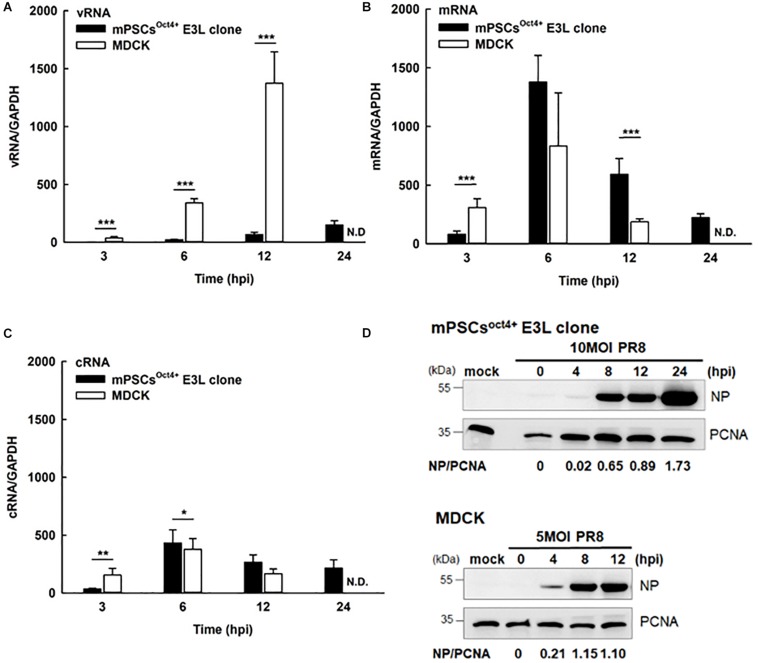
Influenza life cycles in the mouse pulmonary stem/progenitor cells (mPSCs)^Oct4+^ E3L clone and Madin-Darby canine kidney (MDCK) cells. Quantification of **(A)** viral RNA (vRNA), **(B)** mRNA, and **(C)** complementary RNA (cRNA). The amounts of vRNA, mRNA, and cRNA in the PR8-infected mPSCs^Oct4+^ E3L clone and MDCK cells were determined by real-time PCR. The relative ratio of each time point were first normalized to its internal control *GAPDH* and then normalized to 0 hpi. At least three independent experiments were performed, and the ratios were presented as mean ± SD. N.D., none detected; ^∗^*p* < 0.05; ^∗∗^*p* < 0.01; ^∗∗∗^*p* < 0.001. **(D)** Nucleoprotein (NP) protein expression in influenza virus-infected mPSCs^Oct4+^ E3L clone and MDCK cells. The mPSCs^Oct4+^ E3L clone and MDCK cells were infected by PR8 at a multiplicity of infection (MOI) of 10. The cell lysates were harvested at 0, 4, 8, and 12 hpi, and the NP expression was determined by the Western blot. The expression of PCNA was used as the input control.

**FIGURE 6:**
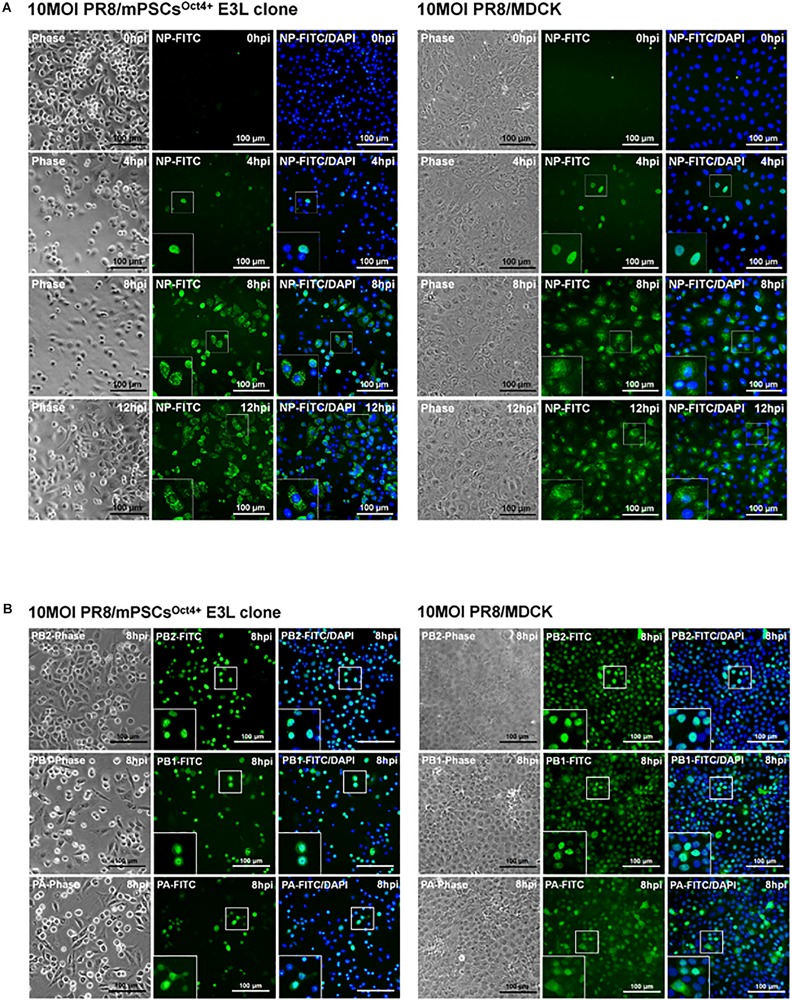
Intracellular distribution of influenza virus nucleoprotein (NP), PA, PB1, and PB2 proteins in the mouse pulmonary stem/progenitor cells (mPSCs)^Oct4+^ E3L clone and Madin-Darby canine kidney (MDCK) cells. **(A)** The distribution of influenza virus NP proteins in the mPSCs^Oct4+^ E3L clone and MDCK cells. The distribution of influenza virus NP proteins in the mPSCs^Oct4+^ E3L clone and MDCK cells at 0, 4, 8, and 12 hpi was determined by immunofluorescence assay (IFA). **(B)** The expression of PA, PB1, and PB2 proteins in the mPSCs^Oct4+^ E3L clone and MDCK cells. The expression of PA, PB1, and PB2 proteins in the mPSCs^Oct4+^ E3L clone and MDCK cells at 8 hpi was determined by IFA, respectively. Scale bar was 100 μm.

Life cycle of influenza viruses in the mPSCs^Oct4+^ E3L clone was characterized to determine the plausible reasons for reduced virus replication in E3L clones as compared to MDCK cells at low virus input. First, binding and entry assays were performed to determine whether the differential virus replication was attributed to reduced viral entry. Nevertheless, a significant increased binding (38.8 vs. 31.9%, *P* = 0.007) and penetration (51.8 vs. 39.4%, *P* < 0.001) were observed in the E3L clone compared to MDCK cells (Supplementary Table S2). There was no difference in entry between these two cells (52.39 vs. 48.76%, *P* = 0.21). Next, we aimed to characterize the profiles of three viral RNA species in the E3L clones. In the early stage of infection (3 hpi), expression of three viral RNA species was higher in MDCK cells than those in mPSCs^Oct4+^. However, higher mRNA and cRNA expression was observed in mPSCs^Oct4+^ than in MDCK cells at 6 hpi (Figures 5B,C), yet vRNA expression in mPSCs^Oct4+^ remained lower than that of MDCK during the study period (Figure 5A). According to previous studies, unlike mRNA and cRNA whose replication depends on the original polymerase complexes on the imported vRNP, yet vRNA replication requires newly synthesized polymerase complexes (Jorba et al., 2009; York et al., 2013). Therefore, expression of the major vRNP proteins, NP, was determined. Although a higher expression of NP was detected at 4 hpi in MDCK than that in the mPSCs^Oct4+^ E3L clone, no significant difference between the E3L clone and MDCK cells was observed at 8 and 12 hpi (Figure 5D). Next, the distribution of viral proteins PB2, PB1, PA, and NP in infected cells was investigated by IFA. The expression of NP was mainly restricted in the nucleus at 4 hpi in both MDCK cells and the E3L clone. However, at 8 hpi, a unique aggregation of NP proteins was observed in the cytosol of the E3L clone, and such pattern remained at 12 hpi (Figure 6A). Furthermore, aggregation of NP proteins was also observed in the cytosol of the mPSCs at 12 hpi (Supplementary Figure S7). Unlike NP proteins, the distributions of influenza polymerase complex proteins, PB2, PB1, and PA, in both MDCK cells and E3L clones were similar and mainly restricted in the nucleus (Figure 6B).

### Cytokine and Chemokines Released From mPSCs^Oct4+^ After PR8 Infection

**FIGURE 7:**
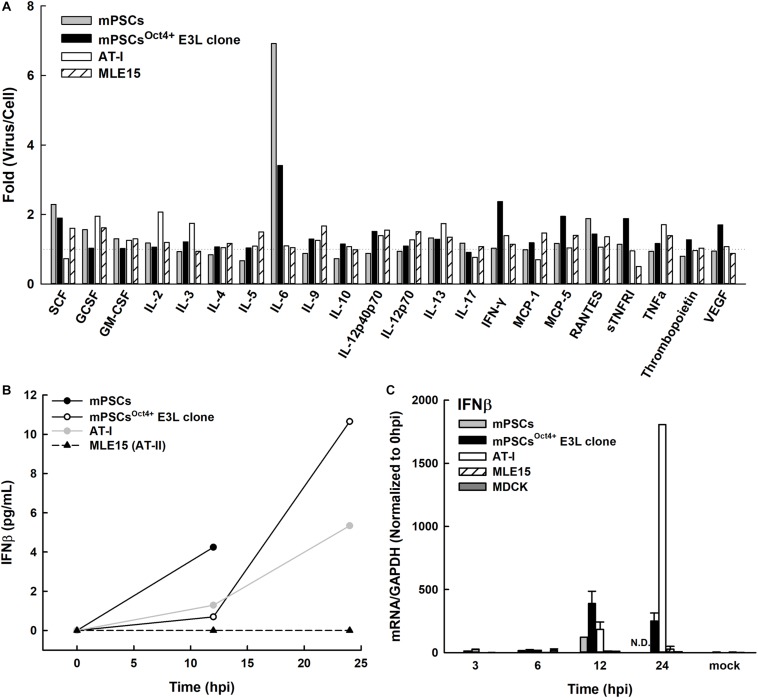
Profiles of releasing cytokines and chemokines from PR8-infected mouse pulmonary stem/progenitor cells (mPSCs), mPSCs^Oct4+^ E3L clone, AT-I, and MLE15 cells. **(A)** Fold changes of cytokines and chemokines released from PR8-infected cells. Cells were infected with PR8 at a multiplicity of infection (MOI) of 10. The cultured supernatants were harvested from mock-infected or PR8-infected cells at 12 hpi. Profiles of releasing cytokines and chemokines in the cultured supernatants were determined by the cytokine array. The fold changes of each cytokine or chemokine between virus- and mock-infected cells were shown. **(B)** Release of interferon (IFN)-β from the PR8-infected mPSCs, mPSCs^Oct4+^ E3L clone, AT-I, and MLE15 cells. The cells were infected with PR8 at an MOI of 10. Concentration of IFN-β in the culture supernatants collected at 0, 12, 24, 40, or 48 hpi was determined by ELISA. **(C)** Induction of IFN-β mRNA expression in the mPSCs, mPSCs^Oct4+^ E3L clone, AT-I, MLE15, and Madin-Darby canine kidney (MDCK) cells after PR8 infection. Total RNA was extracted from PR8-infected cells at 0, 3, 6, 12, and 24 hpi. mRNA of IFN-β and GAPDH was quantified by RT-PCR. The expression of IFN-β mRNA was normalized with GAPDH mRNA. The ratios shown were normalized to 0 hpi. At least three independent experiments were performed, and the ratios were presented as mean ± SD. N.D., none detected.

Upon influenza virus infection, the airway epithelial cells would initiate a rapid onset of the innate immune responses to prevent virus infection. The profiles of cytokines and chemokines released in influenza virus-infected mPSCs, mPSCs^Oct4+^ E3L clone, mPSCs-differentiated AT-I and MLE15 cells were determined at 12 hpi. Compared to the mock group, more than 1.5-fold increase of protein expression of IL-6, IFN-γ, MCP-5, SCF, sTNFRI, VEGF, and IL12p40/p70 was observed in E3L clone (Figure 7A). The intensely enhanced expression of IL-6 was also observed in mPSCs and mPSCs^Oct4+^ E3L clone. Additionally, a more than 1.5-fold increase of SCF and GCSF was also detected in mPSCs, mPSCs^Oct4+^ E3L clone, and MLE15 cells. The IFN-β protein levels in the culture supernatants of influenza-infected mPSCs, mPSCs^Oct4+^ E3L clone, AT-I, and MLE15 cells were also examined by ELISA (Figure 7B). A drastic increase of IFN-β from 0.69 pg/ml at 12 hpi to 10.65 pg/ml at 24 hpi was observed in the virus-infected mPSCs^Oct4+^ E3L clone. The increase of IFN-β in virus-infected mPSCs and AT-I cells was also observed. However, no IFN-β expression was detected in virus-infected MLE15 cells. Furthermore, IFN-β mRNA expression was determined by RT-PCR (Figure 7B). Compared to virus-infected MDCK cells, mRNA expressions of IFN-β in mPSCs, E3L clone, and AT-I cells were similar at the early stage (3 and 6 hpi) but increased significantly at later stage (12 hpi). Consistent to the results of IFN-β protein level, mRNA expression of IFN-β was not increased in virus-infected MLE15 from 0 to 24 hpi (Figure 7C).

## Discussion

Previously, progenitor cells isolated from PR8-infected C57BL/6 mice lung were shown to be more susceptible to PR8 infection than AT-I, AT-II, and bronchial epithelial cells (Quantius et al., 2016). In addition, these virus-infected stem/progenitor cells tend to lose their ability to repair damage due to blockage of Fgfr2b-dependent renewal through inhibiting β-catenin-mediated transcription (Quantius et al., 2016). It was also demonstrated that Oct4^+^ lung stem/progenitor cells from different species could be infected by human H1N1 influenza, swine H1N1 influenza, and avian H7N2 influenza viruses (Khatri et al., 2012). These findings implicate that lung/progenitor cells could be infected by influenza viruses and might play a role in influenza virus-induced pathogenesis. In this study, Oct4^+^Sca1^+^SSEA1^+^ mPSCs were isolated from newborn ICR mice to examine their susceptibility to influenza virus infection, and these cells were further immortalized by transducing mouse *Oct4* for subsequent *in vitro* analysis. We demonstrated that these mPSCs^Oct4+^ cells are susceptible to influenza virus infection and can release similar profiles of cytokines/chemokines after viral infection. In addition, due to the demonstrated expressions of cytokeratin-7 and peroxiredoxin 2 and 6 and relatively high level of CyP450 activities in previous study (Gu et al., 2016), our mPSCs might be closely associated with the lineage of club cells, which were reported as target cells of influenza virus infection, and a significant loss of such cells in C56BL/6 mouse infected with influenza viruses has been reported (Kumar et al., 2011; Hirano et al., 2016). These results implicate that our mPSCs could serve as *in vitro* target cells for influenza A viruses to modulate early pro-inflammatory responses to influenza virus infection.

In this study, the profile of growth factors, cytokines, and chemokines released from influenza virus-infected mPSCs^Oct4+^ was determined. Previously, increased levels of MCP-1, IL-6, TNF-α, IFN-γ, IL-5, IL-4, and IL-9 in bronchoalveolar lavage fluid (BALF) from PR8-challenged C57BL/6 mice have been reported (Buchweitz et al., 2007). The increased expression of MCP-1, IL-6, TNF-α, RANTES, KC, and MIP2 was detected in primary airway epithelial cells isolated from C57BL/6 mice (Tate et al., 2011). Similar to previous studies, our study results showed that compared with uninfected cells, increased levels of IL-6 and IFN-γ as well as IL12p40p70, MCP-5, SCF, sTNFRI, RANTES, and VEGF were observed at 12 hpi. Elevated expression of IFN-β was also determined by ELISA at 12 and 24 hpi. Interestingly, increased levels of anti-inflammatory cytokines soluble tumor necrosis factor receptor I (sTNFRI) were detected in PR8-infected mPSCs^Oct4+^. sTNFRI was shown in previous studies to interfere with the interactions between TNF-α and its membrane form receptor (mTNFR) and subsequently lead to downregulation of TNF-α-induced inflammation (Levine, 2008). Such elevated levels of sTNFRI were also found in mouse model (Lowder et al., 2006; Gabriel et al., 2009) or human (Hoffmann et al., 2015) after H1N1 or H7N9 influenza virus infection, respectively. An increased level of VEGF in cultured supernatants of PR8-infected mPSCs^Oct4+^ was also noticed. VEGF, as a vascular permeability factor, plays a crucial role in lung maintenance, inducing proliferation of systemic vasculature, stimulating growth of AT-II, and secreting surfactant protein (Barratt et al., 2014). VEGF also has chemotaxis ability to attract alveolar macrophages, which express VEGF receptor 2 (VEGFR2) (Clauss et al., 1996; Ferrara et al., 2003). The increased expression of cytokines in PR8-infected mPSCs^Oct4+^ might implicate that mPSCs play an important role as immune sensor upon PR8 infection to attract infiltration of immune regulatory cells.

To better characterize virus replication in mPSCs^Oct4+^, series of assays were performed in this study. Compared with the commonly used cell line MDCK, similar trend of virus growth was observed, albeit with a relatively lower virus propagation in PR8-infected mPSCs and mPSCs^Oct4+^ cells. A significantly higher percentage of virus binding (38.82 vs. 31.9%, *P* = 0.00685) and penetration (51.75 vs. 39.41%, *P* = 0.00024) was observed in PR8-infected mPSCs^Oct4+^ as compared to MDCK cells. However, the entry percentages were similar (52.39 vs. 48.76%, *P* = 0.21322) between PR8-infected mPSCs^Oct4+^ and MDCK cells. The inconsistent results of binding/penetration and entry ability might indicate that virus envelope uncoating, vRNP importing into nucleus, or even RNA replication is faster in MDCK cells than mPSCs^Oct4+^. To investigate the difference of viral RNA replication and transcription efficiency between these two cells, expressions of viral vRNA, cRNA, and mRNA were quantified by RT-PCR. A more dramatic difference in viral vRNA replication in mPSCs^Oct4+^ at 6 and 12 hpi was observed (Figure 5A). It was recently reported that viral vRNA and cRNA replication are initiated by soluble polymerase complexes in *trans*, but mRNA replication is initiated by resident polymerase complexes in *cis* (Eisfeld et al., 2015). Although vRNA and cRNA both are negative stranded RNA and are synthesized by the same polymerase complexes, different promoter sequences are used to initiate RNA replication (Azzeh et al., 2001). Previous study indicated that p65, one component in the NF-κB, was involved in regulating vRNA synthesis from the cRNA promoter (Kumar et al., 2008). NF-κB signaling can be activated by pro-inflammatory cytokines, such as TNFα, IL-1, or toll-like receptors (Lawrence, 2009), and plays a role in regulating the expression of cytokines, chemokines, or adhesion molecules. Therefore, high expression of sTNFRI in culture supernatant of PR8-infected mPSCs^Oct4+^ might downregulate TNF-α, activating NF-κB signaling pathway, and subsequently lead to impairment in vRNA replication.

Quiescence nature of embryonic stem cells might result in restricted viral protein translation and subsequent defeat viral protein production (Wash et al., 2012). In our study, we found that, compared to MDCK, less NP expression in the influenza-infected mPSCs^Oct4+^ E3L clone was detected at early time point (4 hpi), yet similar viral protein expression between two cells was observed later at 8 and 12 hpi. Nevertheless, a unique distribution pattern of NP proteins was observed in the influenza-infected mPSCs^Oct4+^ E3L clone. Compared to MDCK, aggregated NP was observed in the cytosol of the mPSCs^Oct4+^ E3L clone at 8 and 12 hpi. Previous study has shown that drug-induced NP aggregation in cytosol might block migration of NP into nucleus and subsequently impair virus replication (Kao et al., 2010). Host protein phospholipid scramblase 1 (PLSCR1) (Luo et al., 2018) and moloney leukemia virus 10 (MOV10) (Zhang et al., 2016) also have been reported can interact with importin α to inhibit viral vRNP imported into nucleus. Therefore, aggregation of NP in mPSCs^Oct4+^ might block vRNP nuclear import, then interfere with vRNA replication. Otherwise, vRNP imports into nucleus were also controlled by the phosphorylation status of NP (Zheng et al., 2015). In that study, the authors found that phosphorylation of S9 and Y10 on nuclear location signal (NLS) blocked the interaction between NP and importin α, causing vRNP retention in the cytosol. However, the possible mechanism of NP aggregation in the cytosol of mPSCs^Oct4+^ needs more studies.

In summary, the mPSCs^Oct4+^ provides a cell model to understand influenza virus infection in mPSCs. This immortalized mPSC cell line can be infected by mouse-adapted PR8 strain, 2009 pandemic H1N1, 2009 seasonal H1N1, 2016 pandemic H1N1, and 2013 avian H7N9 influenza viruses. Impaired vRNA replication and NP aggregation in cytosol might be correlated with lower virus propagation. The investigation of releasing cytokines might indicate the potential role of PR8-infected mPSCs^Oct4+^ as an immune sensor.

## Data Availability Statement

The raw data supporting the conclusions of this article will be made available by the authors, without undue reservation, to any qualified researcher.

## Author Contributions

T-LC, P-HL, and Y-TC performed the virus infection experiments. S-YG was mainly responsible for generating the mPSCs and relative clone selection. S-YC and T-YL supervised the experimental design and participated in the discussion. T-LC, T-YL, and S-YC drafted the manuscript. All authors read and approved the final manuscript.

## Conflict of Interest

The authors declare that the research was conducted in the absence of any commercial or financial relationships that could be construed as a potential conflict of interest.

## Supplementary Material

FIGURE S1 Effects of different virus input in the development of cytopathic effects (CPE) in mPSCs. mPSCs were infected with PR8 at three different MOIs, 10, 1, and 0.1. The morphology changes of the same colony between 0 and 36 hpi were recorded by microscope with a scale bar of 100 μm.

FIGURE S2 Detection of influenza virus release from infected mPSCs, mPSCs^Oct4+^ E3L clone, and MDCK cells by transmission electron microscope (TEM). The mPSCs, mPSCs^Oct4+^ E3L clone, and MDCK cells were infected with PR8 at an MOI of 10. After 12 h post infection, the cells were harvested for TEM analysis. The observation of cell morphology under 8,000x, 30,000x and 80,000x magnification was demonstrated. N, cell nuclear. Cy, Cytosol. Scale bars were 2 μm, 0.5 μm, and 200 nm, respectively. The black arrow indicates virus particles. The black arrow head indicates the viruses with abnormal morphology.

FIGURE S3 Susceptibility of mPSCs-differentiated AT-I and AT-II cell line MLE15 cells to influenza virus infection. **(A)** Infection of AT-I cells by influenza virus. AT-I cells were infected with PR8 at an MOI of 10. The CPE was recorded by microscope with a scale bar of 100 μm. The expression of viral NP proteins in AT-I cells at different time points after virus infection was determined by IFA. Scale bar was 100 μm. **(B)** Infection of MLE15 cells by influenza virus. MLE15 cells were infected with PR8 at an MOI of 10. The CPE was recorded by microscope with a scale bar of 100 μm. The expression of viral NP proteins in MLE15 cells at different time points after virus infection was determined by IFA. Scale bar was 100 μm. **(C)** Replication of influenza virus in AT-I and MLE15 cells. Cultured supernatants were collected at indicated time points and virus titers in the cultured supernatants of AT-I and MLE15 cells were quantified by plaque assay. At least three independent experiments were performed and the virus titers were presented as mean ± SD.

FIGURE S4 The expression of α2,3-linked sialic acid (α2,3 SA) and α2,6-linked sialic acid (α2,6 SA) on mPSCs^Oct4+^ E3L clone after serial passages. The expression of α2,3 SA and α2,6 SA in mPSCs^Oct4+^ E3L clone after 5, 12, and 20 passages was determined by FACS. The histogram of α2,3 SA and α2,6 SA expression were shown in purple, and the negative-staining cells were labeled as green lines.

FIGURE S5 Characterization of virus particles in the supernatants of influenza infected mPSCs^Oct4+^ E3L clone and MDCK cells. **(A)** Purification of virus particles in the supernatants of influenza infected mPSCs^Oct4+^ E3L and MDCK cells. Culture supernatants of virus-infected cells were harvested at 36 hpi and purified on the linear sucrose gradient (20–60% w/v) by ultracentrifugation. The gray dot line indicates the sucrose density (g/mL) in each fraction. The black line indicates infectious virus titer (PFU/mL) determined by the plaque assay. **(B)** Detection of virus proteins in the fractions after ultracentrifugation. The presence of HA and NP proteins in fractioned samples were determined by western blot.

FIGURE S6 Expression of viral NP proteins in influenza virus infected mPSCs^Oct4+^ E3L clone. The mPSCs^Oct4+^ E3L clone was infected with four human influenza virus strains, A/California/07/2009 (H1N1), A/Taipei/0056/2016(H1N1)-like virus and A/Taiwan/S02076/2013 (H7N9) at an MOI of 10. The expression of viral NP proteins in the mPSCs^Oct4+^ E3L clone at 12 hpi was determined by IFA. Scale bar was 100 μm.

FIGURE S7 Intracellular distribution of influenza virus NP protein in mPSCs. mPSCs were infected with PR8 at an MOI of 10. The distribution of viral NP proteins in mock- and PR8-infected cells at 12 hpi were determined by IFA. Scale bar was 100 μm.

TABLE S1 Primer list.

TABLE S2 The percentages of virus binding, penetration and entry on or into the mPSCs^Oct4+^ E3L clone and MDCK cells.
