# Supplementary figures and images for: Characterization of Influenza A Virus Infection in Mouse Pulmonary Stem/Progenitor Cells
Source: Front Microbiol. 2020 Jan 21;10:2942. doi: 10.3389/fmicb.2019.02942 (PMC6985155; doi:10.3389/fmicb.2019.02942)

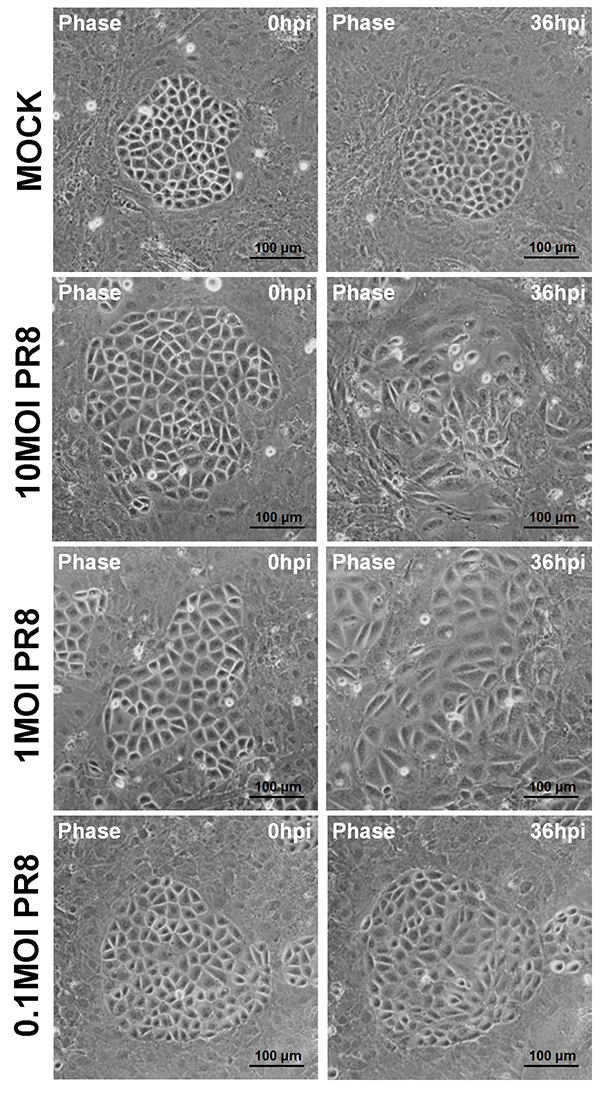

Supplement: FIGURE S1 — Effects of different virus input in the development of cytopathic effects (CPE) in mPSCs. mPSCs were infected with PR8 at three different MOIs, 10, 1, and 0.1. The morphology changes of the same colony between 0 and 36 hpi were recorded by microscope with a scale bar of 100 μm. [file Image_1.TIF]

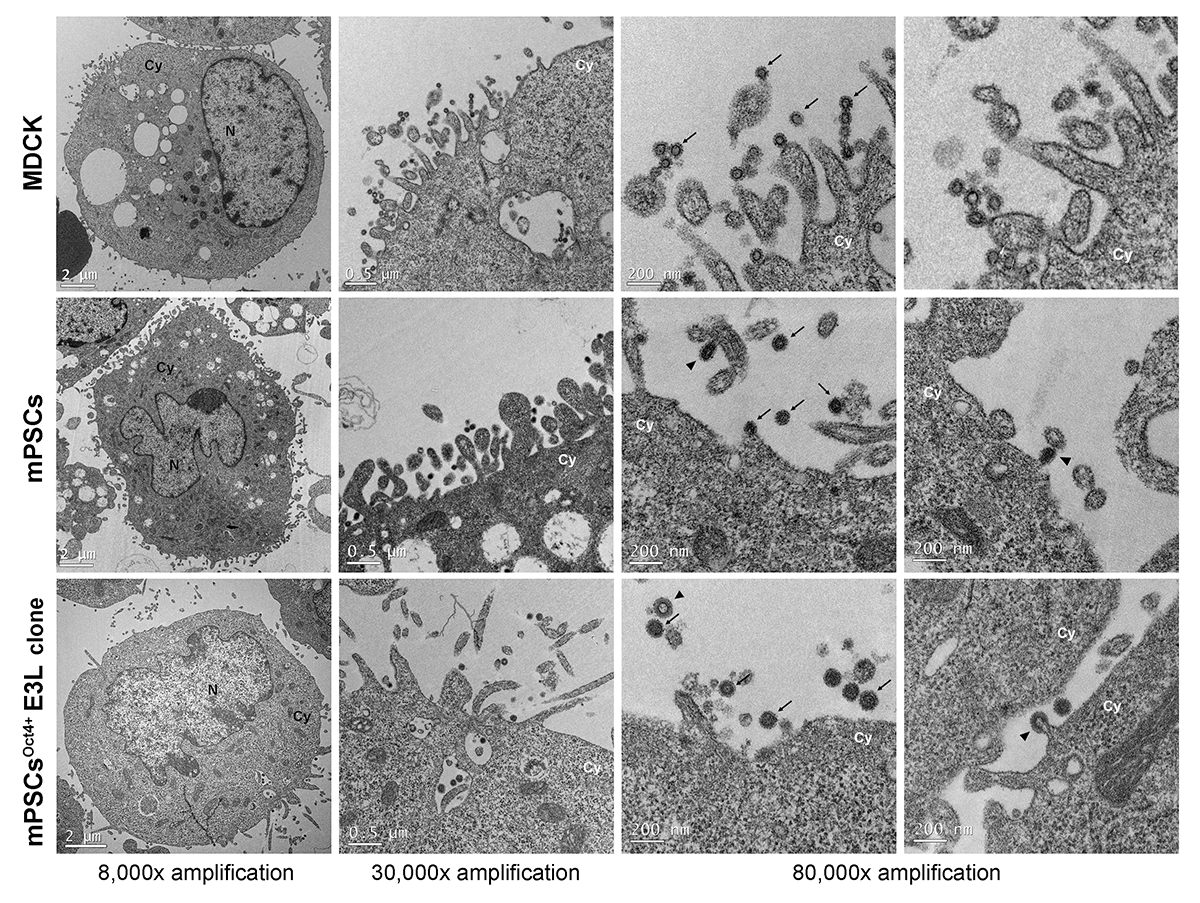

Supplement: FIGURE S2 — Detection of influenza virus release from infected mPSCs, mPSCsOct4+ E3L clone, and MDCK cells by transmission electron microscope (TEM). The mPSCs, mPSCsOct4+ E3L clone, and MDCK cells were infected with PR8 at an MOI of 10. After 12 h post infection, the cells were harvested for TEM analysis. The observation of cell morphology under 8,000x, 30,000x and 80,000x magnification was demonstrated. N, cell nuclear. Cy, Cytosol. Scale bars were 2 μm, 0.5 μm, and 200 nm, respectively. The black arrow indicates virus particles. The black arrow head indicates the viruses with abnormal morphology. [file Image_2.TIF]

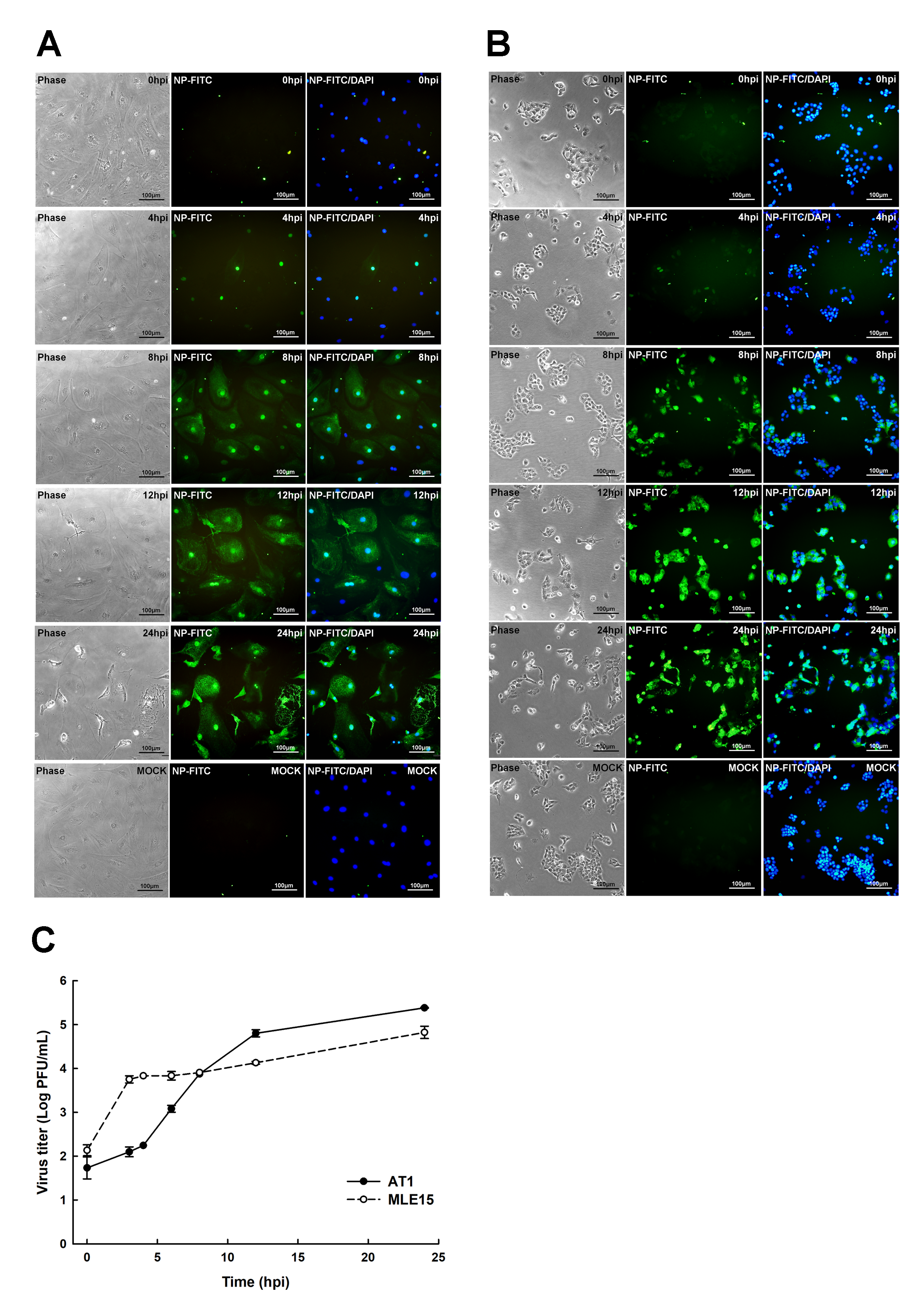

Supplement: FIGURE S3 — Susceptibility of mPSCs-differentiated AT-I and AT-II cell line MLE15 cells to influenza virus infection. (A) Infection of AT-I cells by influenza virus. AT-I cells were infected with PR8 at an MOI of 10. The CPE was recorded by microscope with a scale bar of 100 μm. The expression of viral NP proteins in AT-I cells at different time points after virus infection was determined by IFA. Scale bar was 100 μm. (B) Infection of MLE15 cells by influenza virus. MLE15 cells were infected with PR8 at an MOI of 10. The CPE was recorded by microscope with a scale bar of 100 μm. The expression of viral NP proteins in MLE15 cells at different time points after virus infection was determined by IFA. Scale bar was 100 μm. (C) Replication of influenza virus in AT-I and MLE15 cells. Cultured supernatants were collected at indicated time points and virus titers in the cultured supernatants of AT-I and MLE15 cells were quantified by plaque assay. At least three independent experiments were performed and the virus titers were presented as mean ± SD. [file Image_3.TIF]

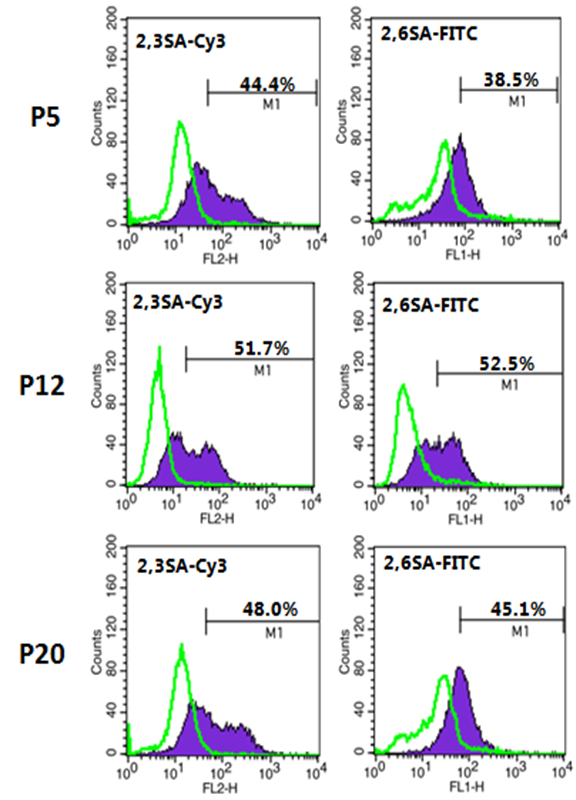

Supplement: FIGURE S4 — The expression of α2,3-linked sialic acid (α2,3 SA) and α2,6-linked sialic acid (α2,6 SA) on mPSCsOct4+ E3L clone after serial passages. The expression of α2,3 SA and α2,6 SA in mPSCsOct4+ E3L clone after 5, 12, and 20 passages was determined by FACS. The histogram of α2,3 SA and α2,6 SA expression were shown in purple, and the negative-staining cells were labeled as green lines. [file Image_4.TIF]

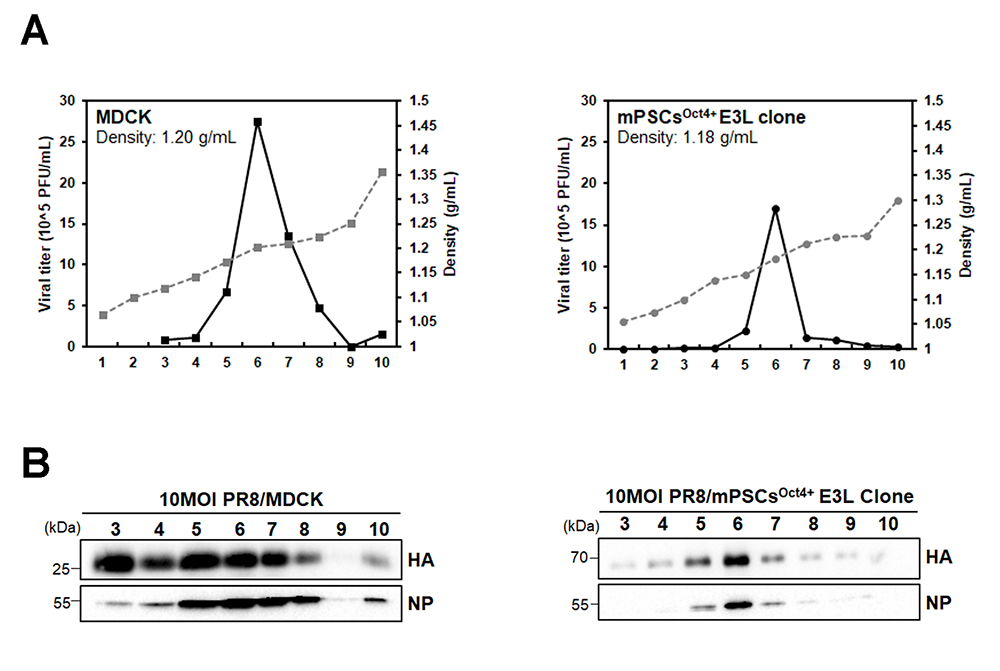

Supplement: FIGURE S5 — Characterization of virus particles in the supernatants of influenza infected mPSCsOct4+ E3L clone and MDCK cells. (A) Purification of virus particles in the supernatants of influenza infected mPSCsOct4+ E3L and MDCK cells. Culture supernatants of virus-infected cells were harvested at 36 hpi and purified on the linear sucrose gradient (20–60% w/v) by ultracentrifugation. The gray dot line indicates the sucrose density (g/mL) in each fraction. The black line indicates infectious virus titer (PFU/mL) determined by the plaque assay. (B) Detection of virus proteins in the fractions after ultracentrifugation. The presence of HA and NP proteins in fractioned samples were determined by western blot. [file Image_5.TIF]

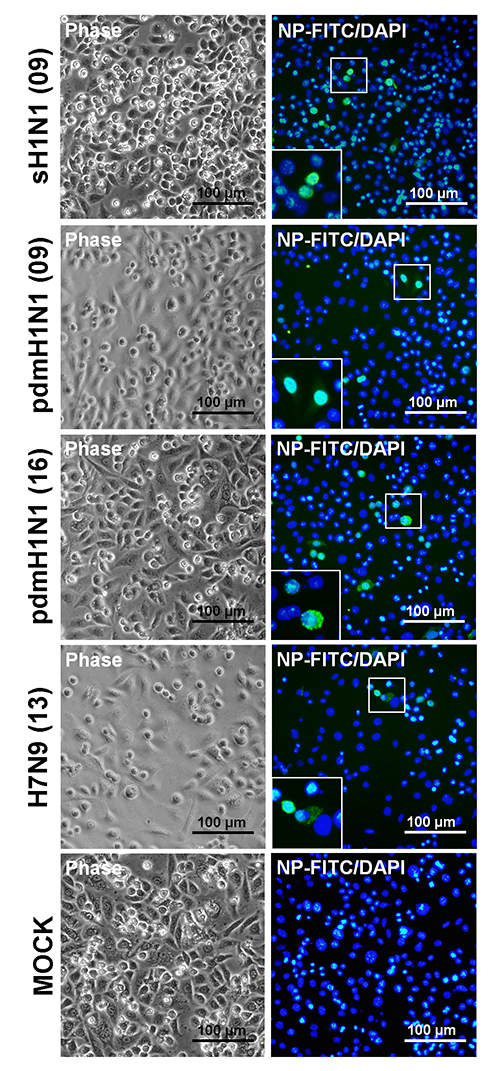

Supplement: FIGURE S6 — Expression of viral NP proteins in influenza virus infected mPSCsOct4+ E3L clone. The mPSCsOct4+ E3L clone was infected with four human influenza virus strains, A/California/07/2009 (H1N1), A/Taipei/0056/2016(H1N1)-like virus and A/Taiwan/S02076/2013 (H7N9) at an MOI of 10. The expression of viral NP proteins in the mPSCsOct4+ E3L clone at 12 hpi was determined by IFA. Scale bar was 100 μm. [file Image_6.TIF]

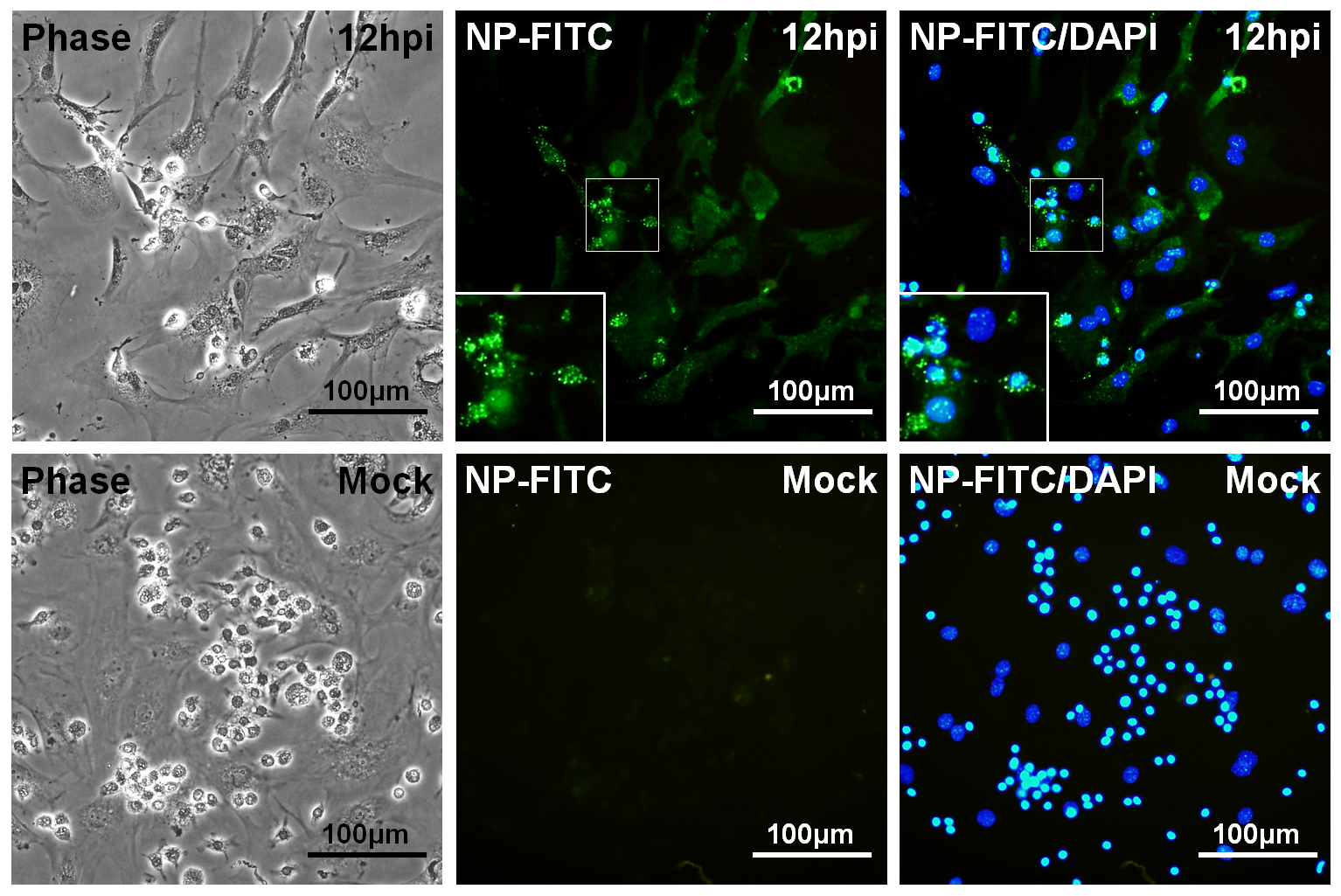

Supplement: FIGURE S7 — Intracellular distribution of influenza virus NP protein in mPSCs. mPSCs were infected with PR8 at an MOI of 10. The distribution of viral NP proteins in mock- and PR8-infected cells at 12 hpi were determined by IFA. Scale bar was 100 μm. [file Image_7.TIF]
