# Supplementary material for: Characterization of Influenza A Virus Infection in Mouse Pulmonary Stem/Progenitor Cells
Source: Front Microbiol. 2020 Jan 21;10:2942. doi: 10.3389/fmicb.2019.02942 (PMC6985155; doi:10.3389/fmicb.2019.02942)
Supplement: TABLE S1 — Primer list. [file Table_1.docx]

| **Supplementary Table 1. Primer list** | | | | |
| --- | --- | --- | --- | --- |
| **Assay** | **Target** | **Primer sequence (5' to 3')** | **Product size** | **Annealing temp (℃)** |
|  | Influenza NP vRNA | 5'-GGC CGT CAT GGT GGC GAA TGA ATG GAC GAA AAA CAA GAA TTG C-3' |  |  |
|  | Influenza NP mRNA | 5'-CCA GAT CGT TCG AGT CGT TTT TTT TTT TTT TTT TCT TTA ATT GTC-3' |  |  |
|  | Influenza NP cRNA | 5'-GCT AGC TTC AGC TAG CGA TCA GTA GAA ACA AGG GTA TTT TTC TTT-3' |  |  |
| PCR | mouse Oct4 | F: 5'-ATG GCT GGA CAC CTG GCT TC-3' | 1121bp | 62 |
|  |  | R: 5'-CCA GGT TCT CTT GTC TAC CTC-3' |  |  |
|  | mouse Sox2 | F: 5'-TAG AGC TAG ACT CCG GGC GAT GA-3' | 297bp | 62 |
|  |  | R: 5'-TTG CCT TAA ACA AGA CCA-3' |  |  |
|  | mouse Nanog | F: 5'-AAA GGA TGA AGT GCA AGC GGT GG-3' | 520bp | 58 |
|  |  | R: 5'-CTG GCT TTG CCC TGA CTT TAA GC-3' |  |  |
|  | mouse Nkx2.1 | F: 5'-GAT CCC GCT CGC ATG TGA TA-3' | 305bp | 60 |
|  |  | R: 5'-GCT TCA ATC CTG TGA CCC CA-3' |  |  |
|  | mouse Id2 | F: 5'-ATG ATC GTC TTG CCC AGG TG-3' | 291bp | 60 |
|  |  | R: 5'-AGC ATT CAG TAG GCT CGT GT-3' |  |  |
|  | mouse SPC | F: 5'-ATT ACT CGG CAG GTC CCA GGA GCC A-3' | 537bp | 58 |
|  |  | R: 5'-AGA TAT AGT AGA GTG GTA GCT CTC C-3' |  |  |
|  | mouse E-Cadherin | F: 5'-CCC AAG TCC AAG ATT TCC ATC C-3' | 781bp | 59 |
|  |  | R: 5'-AAA GCC TCC AGC AAG CAC G-3' |  |  |
| PCR/Real-time PCR | mouse GAPDH | F: 5'-ACC ACA GTC CAT GCC ATC AC-3' | 452bp | 58/60 |
|  |  | R: 5'-TCC ACC ACC CTG TTG CTG TA-3' |  |  |
| Real-time PCR | canine GADPH | F: 5'-CAA CGG ATT TGG CCG TAT TGG-3' | 89bp | 60 |
|  |  | R: 5'-TGA AGG GGT CAT TGA TGG CG-3' |  |  |
|  | mouse IFNβ | F: 5'-TCC AGC TCC AAG AAA GGA CG-3' | 123bp | 60 |
|  |  | R: 5'-GCA TCT TCT CCG TCA TCT CC-3' |  |  |
|  | canine IFNβ | F: 5'-CCA GTT CCA GAA GGA GGA CA-3' | 200bp | 60 |
|  |  | R: 5'-TGT CCC AGG TGA AGT TTC C-3' |  |  |
|  | Influenza NP vRNA | F: 5'-GGC CGT CAT GGT GGC GAA T-3' | 190bp | 60 |
|  |  | R: 5'-CTC AAT ATG AGT GCA GAC CGT GCT-3' |  |  |
|  | Influenza NP mRNA | F: 5'-CGA TCG TGC CTT CCT TTG -3' | 94bp | 60 |
|  |  | R: 5'-CCA GAT CGT TCG AGT CGT-3' |  |  |
|  | Influenza NP cRNA | F: 5'-CGA TCG TGC CTT CCT TTG -3' | 104bp | 60 |
|  |  | R: 5'-GCT AGC TTC AGC TAG GCA TC-3' |  |  |
