# Supplementary material for: Characterization of Influenza A Virus Infection in Mouse Pulmonary Stem/Progenitor Cells
Source: Front Microbiol. 2020 Jan 21;10:2942. doi: 10.3389/fmicb.2019.02942 (PMC6985155; doi:10.3389/fmicb.2019.02942)
Supplement: TABLE S2 — The percentages of virus binding, penetration and entry on or into the mPSCsOct4+ E3L clone and MDCK cells. [file Table_2.docx]

| **Supplementary Table 2. The percentages of virus binding, penetration and entry on or into the mPSCs^Oct4+^ E3L clone and MDCK cells** | | | |
| --- | --- | --- | --- |
|  | Binding (%)^a^* | Penetration (%)^b^* | Entry (%)^c^ |
| mPSCs^Oct4+^ E3L clone | 38.8 (±3.2^d^) | 51.8 (±4.0) | 52.4 (±4.1) |
| MDCK | 31.9 (±3.2) | 39.4 (±2.9) | 48.8 (±4.6) |
| * p-value <0.05  ^a^ The percentage of virus binding was calculated as the copies of binding virus divided by the copies of the input viruses.  ^b^ The percentage of virus penetration was calculated as the copies of penetrated virus divided by the copies of binding viruses.  ^c^ The percentage of virus entry was calculated as the copies of intracellular viruses divided by the copies of input viruses.  ^d^ The values in the brackets were the standard deviations of at least three independent assays. | | | |
